# Sexuality of pregnant, postpartum and breast-feeding women

**DOI:** 10.1192/j.eurpsy.2024.771

**Published:** 2024-08-27

**Authors:** S. Bader, M. aloulou, Z. Zran, A. Abdelmoula, A. Bouaziz, W. Abbes

**Affiliations:** ^1^psychiatry; ^2^obstetric gynecology, Universiry Hospital of Gabes, Gabes, Tunisia

## Abstract

**Introduction:**

Pregnancy and breast-feeding represents a period of psychological maturation for the woman who becomes a mother, a period of significant changes in women’s lives that affects their sexuality and intimacy.

**Objectives:**

To investigate the quality of sexual function in pregnant, postpartum and breastfeeding women.

**Methods:**

It was a cross-sectional study established over a period of 3 months from the June 1^st^, 2023 to August 31, 2023. This study focused on a population of pregnant, postpartum and breastfeeding women recruited from outpatient consultations and inpatient of the obstetric gynecology department at the university hospital of Gabes. We used a pre-established sheet exploring socio-demographic data, medical and gyneco-obstetric history and informations concerning the marital relationship and the woman’s sexual activity. We administered the validated Arabic version of the Arizona Sexual Experiences Scale (ASEX) to assess sexual functioning.

**Results:**

Fifty-eight women were included. The average age was 35.6±5.5 years, they had a university level in 40%, secondary in 37.5%, and they were unemployed in 74.2%. From an urban origin in 75%. They were pregnant in the first, second and third trimester in (15.6%, 15.6% and 25% respectively). They were in postpartum in 43.8% of cases with a cesarean delivery in 73.3% and breastfeeding in 56%. All women reported being on good terms with their spouses and satisfied with their sexuality. The usual frequency of sexual relations (SR) was (1/day: 22.6%, 1/week: 74.2%, 1/month: 3.2%) and 25% reported wanting to reduce the frequency. Only 3.44% masturbated and 5.17% had sexual fantasies. The mean ASEX score was 13 ± 4.3 and 47%of the sample had sexual dysfunction. We found a significant association between the sexual dysfunction and the trimester of pregnancy (p=0.045).Highest score of sexual dysfunction during the first and third trimester compared to the second one (68.9%, 77.6% and 22.4% repectively). The areas of sexual dysfunction were difficulty reaching orgasm (81%), impaired sexual desire (65.5%), insufficient lubrication (60.3%), arousal (55.1%) and pain on penetration (50%).

**Conclusions:**

We found that sexual function is problematic among women during pregnancy especially in the first and third trimester also in postpartum and breastfeeding period. So what factors are associated with this sexual dysfunction?

**Disclosure of Interest:**

None Declared

